# Tests for publication bias are unreliable in case of heteroscedasticity

**DOI:** 10.1016/j.conctc.2021.100781

**Published:** 2021-06-04

**Authors:** Osama Almalik, Zhuozhao Zhan, Edwin R. van den Heuvel

**Affiliations:** a Department of Mathematics and Computer Science, Eindhoven University of Technology, Eindhoven, the Netherlands; b Department of Preventive Medicine and Epidemiology, School of Medicine, Boston University, Boston, USA

**Keywords:** Heteroscedastic mixed effects model, Aggregated data meta-analysis, Mean difference treatment effect sizes

## Abstract

Regression based methods for the detection of publication bias in meta-analysis have been extensively evaluated in literature. When dealing with continuous outcomes, specific hidden factors (e.g., heteroscedasticity) may interfere with the test statistics. In this paper we investigate the influence of residual heteroscedasticity on the performance of four tests for publication bias: the Egger test, the Begg-Mazumdar test and two tests based on weighted regression. In the presence of heteroscedasticity, the Egger test and the weighted regression tests highly inflate the Type I error rate, while the Begg-Mazumdar test deflates the Type I error rate. Although all three tests already have low statistical power, heteroscedasticity typically reduces it further. Our results in combination with earlier discussions on publication bias tests lead us to conclude that application of these tests on continuous treatment effects is not warranted.

## 1 Introduction

In a meta-analysis, publication bias can lead to an incorrect pooled estimate of a treatment effect. In the presence of publication bias, the treatment effect is associated with factors that affect publication bias, e.g., the size of the standard error of the treatment effect or the size of the study. Thus studies with a lack of statistical significance or smaller studies are less likely to be published.

Several methods have been proposed in literature to test for lack of this type of publication bias, e.g., the Egger test [1], the rank-correlation test [2], and several others [1,[3], [4], [5], [6], [7], [8], [9]]. These tests allow for heteroscedastic residual variances of the study effect sizes, but when their performances were studied the underlying data models are typically homoscedastic if sample size differences and realization of standard errors are ignored. We believe that homoscedasticity is not always a valid assumption, especially when dealing with continuous outcomes on individuals. For many clinical and social outcomes the variance may be (inversely) proportional to the mean (e.g., blood pressure in cardiovascular disease [10], forced expiratory volume in respiratory disease [11], heards on dairy sire in genetics [12], smoking-mood relationship in psychology [13], income and consumption in economics [14], grade point average in educations [15], stock market volatility in finance [16], radioimmunoassay in biology [17], pharmacokinetic, enzyme kinetics, and chemical kinetics in pharmacology [18], as well as inequalities in sociology [19]).

In case of heteroscedasticity, studies may or may not follow the premises of the test statistics, but when the variance of the outcome is correlated with its mean the resulting treatment effect estimates will be correlated with its standard errors as well. This may lead to the detection of artificial “publication bias” without the presence of a real publication bias process. Since treatment related heteroscedasticity is not testable with aggregated data in a meta-analysis, there is no guarantee that positive tests results for publication bias are truly positive at all. Therefore, it is crucial to better understand the performances of the publication bias test for aggregated data under heteroscedasticity.

The objective of this paper is to demonstrate that heteroscedasticity negatively affects the performance of test statistics for publication bias in case of continuous outcomes. We will perform a simulation study, since analytical investigations are complicated. We ignore bias adjustment methods, because we expect that correction of pooled estimates for publication bias is even more difficult when the presence or absence of publication bias is hard to determine. The Egger test, the rank-correlation test and two tests based on a weighted regression model [4] are considered. The choice of the Egger test follows from the objective of this paper, since it tests the dependence of the standardized estimated treatment effect with the precision of the study effect size (i.e., the inverse of its standard error). The two weighted regression methods were included because they were recommended for practice in the literature [4], and they might be robust to heteroscedasticity in theory. We decided to also include the rank-correlation test because it is not a regression based method and it is based on the correlation between the normalized treatment effect and its standard error. We did not consider publication bias tests for other outcome types (e.g., binary outcome) or other forms of publication bias (e.g., language bias) [1,3,[5], [6], [7], [8], [9]].

In Section 2 we will describe the four test statistics for publication bias in an aggregated-data meta-analysis. In the same section, we will introduce a heteroscedastic mixed effects model for individual participants in a study [17,20]. This model will be used to formulate the aggregated treatment effect per study and it will be used to simulate data. The third part of Section 2 is about a mechanism of publication bias. This mechanism is based on concepts for study selection and described in literature [1,8,21,22]. Then Section 3 describes a simulation study and the choices of parameter settings. Section 4 presents the simulation results and a discussion is provided in Section 5.

## 2 Methods

### 2.1 Tests for publication bias on aggregated data

An aggregated data meta-analysis usually consists of treatment effect estimates $D_{i}$ obtained from different studies, i (=1,2,...,m), accompanied by their estimated standard errors $S_{i}$ [23]. The four test statistics for testing the hypothesis of no publication bias investigate the association between $D_{i}$ and $S_{i}$.

**Egger's Test:** The Egger test uses a regression model with the standardized effect size $D_{i}/S_{i}$ as response variable and the precision $S_{i}^{-1}$ as the independent variable, respectively. Using a *t*-test, the null hypothesis of no publication bias is rejected if the intercept of the regression model significantly deviates from zero [1].

**Weighted regression:** A weighted regression method uses $D_{i}$ as the response variable, $S_{i}$ as the independent variable and $1/\left(S_{i}^{2}+{\hat{\tau }}^{2}\right)$ as the weight,^1^ with ${\hat{\tau }}^{2}$ the between-study variance estimated with DerSimonian and Laird method [24]. Using a *t*-test, the null hypothesis of no publication bias is rejected if the slope of the independent variable significantly deviates from zero [4]. This method will be referred to as the weighted DL test. However, the DerSimonian-Laird estimate has been found to underestimate the between-study variance estimate, and thereby producing narrow confidence intervals for the mean treatment effect [25]. Instead of the DerSimonian-Laird estimator, the Restricted Maximum Likelihood (REML) estimator have been recommended in literature [26]. We therefore also considered the weighted regression model with $\tau^{2}$ estimated by REML, here referred to as the weighted REML test. We used procedure MIXED in SAS, version 9.4, to calculate this REML estimate ${\hat{\tau }}^{2}$.

**Rank correlation:** The rank-correlation test applies Kendall's tau correlation coefficient to the normalized treatment effect $D_{i}^{\text{*}}=\left(D_{i}-\overset{-}{D}\right)/\sqrt{{\tilde{s}}_{i}^{2}}$ and the variance $S_{i}^{2}$ of the study effect size, with $\overset{-}{D}=\left(\overset{m}{\underset{i=1}{\sum }}D_{i}/S_{i}^{2}\right)/\left(\overset{m}{\underset{i=1}{\sum }}S_{i}^{-2}\right)$ the weighted (fixed) effect size, and ${\tilde{s}}_{i}^{2}=S_{i}^{2}-{\left(\overset{m}{\underset{i=1}{\sum }}S_{i}^{-2}\right)}^{-1}$ the estimated variance of the normalized effect size (under assumption of homogeneity of effect sizes). This non-parametric test statistic follows approximately a standard normal distribution when $D_{i}^{\text{*}}$ and $S_{i}^{2}$ are independent. Thus the associated p-value is used to test the hypothesis of no publication bias [2,8].

### 2.2 Heteroscedastic mixed effects model on individual participants

Let $Y_{ijk}$ denote the continuous response variable of individual k $\left(=1,\cdots ,n_{ij}\right)$, exposed to treatment j (=0,1), in study i (=1,⋯,m). A heteroscedastic linear mixed effects model per individual [20] can then be described as:

(1) $$Y_{ijk}=\mu_{j}+U_{ij}+\xi_{j}exp\left(V_{i}\right)\varepsilon_{ijk},$$

with $\mu_{j}$ the mean of treatment or control group j, $\theta =\mu_{0}-\mu_{1}$ the mean treatment effect, $U_{ij}$ a study-specific random effect for group j, $U_{i0}-U_{i1}$ a random treatment effect for study i, $\xi_{j}^{2}$ a treatment specific residual variance parameter, $V_{i}$ normally distributed, and $\varepsilon_{ijk}\sim N\left(0,1\right)$ standard normally distributed and independent of the random effects $U_{i0}$, $U_{i1}$, and $V_{i}$. Residual heteroscedasticity at the individual level is introduced via parameter $\xi_{j}^{2}$ and the random term $exp\left(V_{i}\right)$. The variance $\xi_{j}^{2}$ indicates a fixed heteroscedasticity in variability between individuals for the two treatment groups (i.e., treatment affects both the level and the variability) and $exp\left(V_{i}\right)$ indicates a random heteroscedasticity between individuals across studies (i.e., individuals are more or less alike within studies). Thus, the random variable $V_{i}$ makes it a heteroscedastic mixed effects model and not the variance parameters $\xi_{j}^{2}$, because these parameters only introduce heteroscedasticity within study. If $V_{i}$ is degenerated in 0, model (1) becomes a simple mixed effects model.

It is assumed that ${\left(U_{i0},U_{i1},V_{i}\right)}^{T}$ has a multivariate normal distribution with means 0 and variance-covariance matrix Σ given by

$$\text{Σ}=\left(\begin{array}{lll} \sigma_{0}^{2} & \rho_{M}\sigma_{0}\sigma_{1} & \rho_{V}\sigma_{0}\sigma_{2}\\ \rho_{M}\sigma_{0}\sigma_{1} & \sigma_{1}^{2} & \rho_{V}\sigma_{1}\sigma_{2}\\ \rho_{V}\sigma_{0}\sigma_{2} & \rho_{V}\sigma_{1}\sigma_{2} & \sigma_{2}^{2} \end{array}\right)$$

The value of $\rho_{M}$ represents the correlation between the study-specific random effects $U_{i0}$ and $U_{i1}$ for the treatment and the control group, respectively. The random treatment effect $U_{i0}-U_{i1}$ represents the study heterogeneity of the study effect size as follows: If $\rho_{M}=1$ and $\sigma_{0}=\sigma_{1}$, $U_{i0}-U_{i1}$ is degenerate in zero or non-existent, while for all other settings of $\rho_{M}<1$, $\sigma_{0}>0$, and $\sigma_{1}>0$ it will lead to study heterogeneity. The value $\rho_{V}$ represents the correlation between the mean and the logarithm of the random heteroscedastic residual variance.

The treatment effect per study is given by the raw mean difference $D_{i}={\overset{-}{Y}}_{i0.}-{\overset{-}{Y}}_{i1.}$ for study i, where ${\overset{-}{Y}}_{ij.}=\overset{n_{ij}}{\underset{k=1}{\sum }}Y_{ijk}/n_{ij}$ is the average value for group j in study i. The standard error $S_{i}$ for the effect size in study i is given by $S_{i}^{2}=S_{i0}^{2}/n_{i0}+S_{i1}^{2}/n_{i1}$, where $S_{ij}^{2}=\overset{n_{ij}}{\underset{k=1}{\sum }}{\left(Y_{ijk}-{\overset{-}{Y}}_{ij.}\right)}^{2}/\left(n_{ij}-1\right)$ is the sample variance for treatment group j in study i. Based on model (1), the treatment effect can be written into the well-known random effects model^2^ for meta-analysis studies [23].

(2) $$D_{i}=\theta +U_{i}+\varepsilon_{i},$$

with $U_{i}=U_{i0}-U_{i1}$, $\varepsilon_{i}=exp\left(V_{i}\right)\left(\xi_{0}{\overset{-}{\varepsilon }}_{i0.}-\xi_{1}{\overset{-}{\varepsilon }}_{i1.}\right)$, and ${\overset{-}{\varepsilon }}_{ij.}=\overset{n_{ij}}{\underset{k=1}{\sum }}\varepsilon_{ijk}/n_{ij}$. Without the existence of $V_{i}$, the residuals $\varepsilon_{i}$ in (2) are homoscedastic if sample sizes $n_{i}$are consistent across studies. The variance $S_{i}^{2}$ can be rewritten into

(3) $$S_{i}^{2}=exp\left(2V_{i}\right)\left(\xi_{0}^{2}s_{i0}^{2}/n_{i0}+\xi_{1}^{2}s_{i1}^{2}/n_{i1}\right),$$

with $\left(n_{ij}-1\right)s_{ij}^{2}=\overset{n_{ij}}{\underset{k=1}{\sum }}{\left(\varepsilon_{ijk}-{\overset{-}{\varepsilon }}_{ij.}\right)}^{2}$ chi-square distributed with $n_{ij}-1$ degrees of freedom.

Clearly, the introduced random heteroscedasticity $V_{i}$ affects both the treatment effect $D_{i}$ and the standard error $S_{i}$. As a consequence, $V_{i}$ affects the responses and the independent variables used in the Egger, the weighted DL, the weighted REML, and the rank correlation test. It also makes an analytical investigation of the test statistics more complex, since the joint distribution of the responses and independent variables are less traceable. We therefore studied the influence of the random residual heteroscedasticity on the three test statistics by simulation.

### 2.3 Publication bias mechanism on aggregated data

We briefly describe the selection model which will create publication bias at study level [5,21]. For each study i∈{1,2,...,m} in the meta-analysis, the selction model assumes a latent variable $Z_{i}$ that depends on the standardized mean difference $D_{i}/S_{i}$. If the latent variable is positive ($Z_{i}>0$), study i is published and appearsin the meta-analysis study, but when it is non-positive ($Z_{i}\leq 0$) the study is not published and may create selection bias in the meta-analysis. The latent variable is given by

(4) $$Z_{i}=\alpha +\beta \cdot D_{i}/S_{i}+\delta_{i},$$

with α and β>0 two constants and $\delta_{i}\sim N\left(0,1\right)$ standard normally distributed and independent of all other random terms. Thus the larger the standardized treatment effect, the larger the probability of being selected (assuming that effect sizes are more frequently positive).

Note that the selection process of studies will be affected by the random residual heteroscedasticity $V_{i}$ through the standardized effect size $D_{i}/S_{i}$. The standardized effect size can be rewritten in

(5) $$D_{i}/S_{i}=\left[\left(\beta +U_{i}\right)exp\left\{-V_{i}\right\}+\nu_{i}e_{i}\right]/\sqrt{\xi_{0}^{2}s_{i0}^{2}/n_{i0}+\xi_{1}^{2}s_{i1}^{2}/n_{i1}},$$

with $\nu_{i}^{2}=\xi_{0}^{2}/n_{i0}+\xi_{1}^{2}/n_{i1}$, $e_{i}\sim N\left(0,1\right)$, and $U_{i}=U_{i0}-U_{i1}$. Thus the difference in behavior of $Z_{i}$ with and without heteroscedasticity is determined by a difference in behavior of $\left(\beta +U_{i}\right)exp\left\{-V_{i}\right\}$ and $\beta +U_{i}$, respectively. The distribution of $\beta +U_{i}$ is symmetric and normal, while $\left(\beta +U_{i}\right)exp\left\{-V_{i}\right\}$ is skewed to the right and non-normal. Combined with the choices for the constant α and β, the probability of selecting a study is lower under heteroscedasticity than under homoscedasticity when these studies would have the same standardized effect size $D_{i}/S_{i}$.

## 3 Simulation study

We simulated a meta-analysis study with m studies and vary the sample size $n_{i}$ for study i=1,⋯,m. This sample size was selected from an overdispersed Poisson distribution, i.e., $n_{i}|\gamma_{i}\sim \text{Poi}\left(\lambda exp\left\{0.5\gamma_{i}\right\}\right)$, with $\gamma_{i}\sim \Gamma \left(a_{0},b_{0}\right)$ drawn from a gamma distribution. Then within each study the participants are randomly allocated to the treatment and the control group with equal probabilities, resulting in $n_{i1}$ participants in the treatment group, and $n_{i0}$ participants in the control group (i.e., $n_{i0}|n_{i}\sim \text{Bin}(n_{i},p$)). The continuous response $Y_{ijk}$ is then simulated according to the heteroscedastic linear mixed effects model described in Section 2.2. The data from this model is then used to calculate the study effect size $D_{i}$ and its standard error $S_{i}$.

To introduce publication bias, a selection process or mechanism is simulated according to the selection model described in Section 2.3. We used the 5% and 95% quantiles of the set of standardized treatment effects $D_{1}/S_{1}$, $D_{2}/S_{2}$, …, $D_{m}/S_{m}$, and denote them by $q_{5}$ and $q_{95}$, respectively. The values α and β are chosen such that $P\left(Z_{i}>0|D_{i}/S_{i}=q_{95}\right)=0.99$ and $P\left(Z_{i}>0|D_{i}/S_{i}=q_{5}\right)=0.025$. Thus relatively small standardized effect sizes, with respect to other studies, will be published with low probability and large standardized effect sizes will be published almost always. That the publication of one study depends on other studies may be reasonable if more research is already known on the same topic. Solving the two probability equations results in $\alpha =\left(-1.96q_{95}-2.33q_{5}\right)/\left(q_{95}-q_{5}\right)$ and $\beta =\left(-1.96-\alpha \right)/q_{5}$, using the normality assumption of the random term $\delta_{i}$ and its independence with the standardized treatment effects. Note that creating α and β in this way results in different values for α and β per simulated meta-analysis.

Different simulation settings were considered both with and without publication bias and with $\left(\sigma_{2}^{2}>0\right)$ and without $\left(\sigma_{2}^{2}=0\right)$ random heteroscedasticity. The settings of the parameters are chosen such that the simulation corresponds approximately with a meta-analysis of clinical trials on for instance hypertension treatment. Parameter settings used to generate the aggregated data $\left(D_{i},S_{i}\right)$ from the individual participant data are m∈{20,50,100}, λ=100, $a_{0}=b_{0}=1$, p=0.5, μ=160, θ=−5, $\xi_{0}^{2}=\xi_{1}^{2}=100$, $\sigma_{0}^{2}=2$, $\sigma_{1}^{2}=3$, $\sigma_{2}^{2}=1$, $\rho_{M}=0.7$ and $\rho_{V}\in \left\{-0.7,-0.5,-0,3,0,0.3,0.5,0.7\right\}$. We will run all combinations of parameter choices and simulate 1000 meta-analysis studies.

Based on the number of studies that remain in the meta-analysis, the four publication bias methods in Section 2.1 were used to test for publication bias. The four test statistics were applied to the same meta-analysis data and they were considered significant at the level of 0.1 in order for our results to be comparable to other studies [4,6,[27], [28], [29], [30]]. We will study the type I error and the power of these tests. We will also report the effective number of studies used in the meta-analyses. The simulation of the meta-analysis data and the analysis of the data was conducted with SAS software, version 9.4.

## 4 Results

The Type I error rate and the power of Egger's test, the rank correlation test (RC), the weighted DL (wDL) and the weighted REML (wREML) test are presented in Table 1 and Table 2, respectively. For the power values, the effective number of studies (as percentage of the number m) ranged from 37.18% to 39.55% on average for $\rho_{V}=-0.7$ to $\rho_{V}=0.7$.

**Table 1:** Type I error rate (%) of the four tests on publication bias.

m	Test	Correlation $\rho_{V}$							σ_2_ = 0
		−0.7	−0.5	−0.3	0	0.3	0.5	0.7	
20	Egger	20.5	19.4	17.4	16.5	16.6	17.0	17.5	18.5
	wDL	14.2	12.7	10.8	10.7	10.0	10.3	10.2	10.3
	wREML	13.9	12.4	10.7	10.2	10.0	10.2	9.9	10.0
	RC	6.4	5.3	5.1	4.5	4.2	5.3	5.1	8.6
50	Egger	25.6	24.5	22.9	22.1	21.7	23.9	27.2	21.4
	wDL	16.0	12.3	11.5	11.7	13.3	13.6	16.9	11.8
	wREML	16.2	12.5	11.3	11.5	12.9	13.6	16.7	12.0
	RC	8.3	7.2	6.1	5.4	5.7	6.8	7.7	9.3
100	Egger	35.0	29.8	27.7	25.7	28.7	31.5	37.0	25.6
	wDL	21.5	15.1	12.1	10.3	13.4	17.0	21.9	10.0
	wREML	21.2	15.4	12.1	10.6	13.3	16.9	22.2	10.4
	RC	10.4	8.5	6.4	6.4	6.7	8.5	11.2	9.3

**Table 2:** Power (%) of the four tests on publication bias.

m	Test	Correlation $\rho_{V}$							σ_2_ = 0
		−0.7	−0.5	−0.3	0	0.3	0.5	0.7	
20	Egger	19.4	20.5	22.3	24.1	25.3	27.5	30.4	25.9
	wDL	18.9	19.1	20.4	22.3	25.1	25.4	26.7	21.8
	wREML	18.6	18.5	20.4	22.0	24.5	25.5	26.3	21.3
	RC	11.7	12.6	13.6	14.9	17.2	19.4	17.9	17.1
50	Egger	30.6	32.6	35.3	38.1	42.8	48.5	52.8	50.0
	wDL	27.8	32.9	35.3	40.0	45.7	50.2	55.4	46.2
	wREML	27.4	32.7	34.4	40.1	45.9	50.4	55.3	45.9
	RC	13.9	17.4	19.3	24.2	26.1	31.1	33.5	34.1
100	Egger	40.7	46.2	51.5	58.5	64.9	68.1	71.7	70.2
	wDL	42.7	48.9	56.2	64.1	72.5	75.9	81.0	69.8
	wREML	42.9	49.3	55.4	63.8	72.4	76.1	81.0	69.8
	RC	24.8	28.5	32.8	40.4	47.7	50.9	56.7	54.1

From Table 1, it can be seen that when heteroscedasticity is absent ($\sigma_{2}=0$), the weighted regression approaches and the rank correlation approach show a nominal Type I error rate of approximately 10% (although the rank correlation test was slightly conservative at m=20). When heteroscedasticity is introduced, the Type I error rates of the Egger test, the weighted DL test and the weighted REML seem to be close to their Type I error rates under homoscedasticity when m=20 and $\rho_{V}\geq -0.3$. However, the Type I error rate increases for these three tests when $\rho_{V}<-0.3$, compared to their Type I error rates at homoscedasticity. When the number of studies m increases we see a different pattern. The Type I error rates of these three tests increase as $\left|\rho_{V}\right|$ increases.

**Fig. 1:**
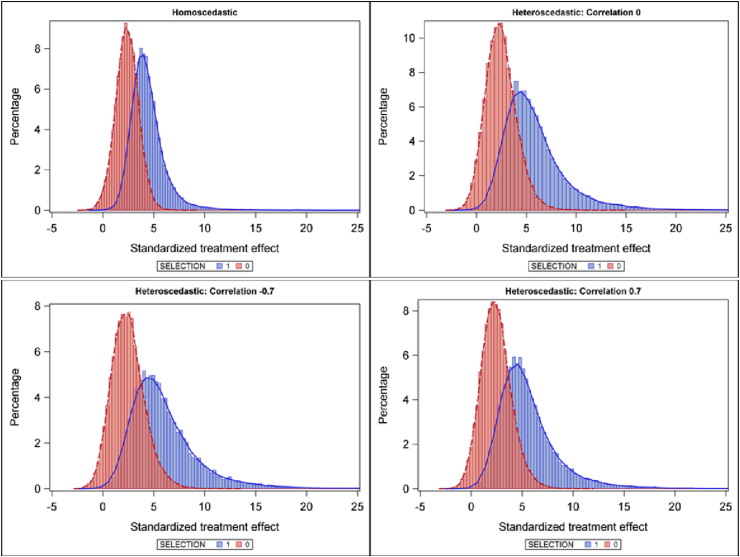
Histograms of the standardized treatment effects under homoscedasticity and three setting of heteroscedasticity. The red histogram represents the studies that are eliminated from the simulated meta-analysis and the blue histograms represent. (For interpretation of the references to colour in this figure legend, the reader is referred to the Web version of this article.)

When publication bias is introduced, heteroscedasticity influences the statistical power of the four test statistics in a different way than for the Type Ierror rates. The power is increasing with the correlation $\rho_{V}$. This would make sense. If the heteroscedasticity is negatively correlated with the heterogeneity of effect sizes ($\rho_{V}<0$), this correlation reduces the positive correlation between study effect sizes and standard errors that is introduced by the publication bias (antagonism). For positive values of $\rho_{V}$ the publication bias is increased (synergism). However, it is somewhat more complicated than just the presence of synergism and antagonism, because the heteroscedasticity also affects the publication bias mechanism (see Fig. 1). Under heteroscedasticity non-selected studies may have higher standardized effect sizes than under homoscedasticity, while selected studies may have lower standardized effect sizes than under homoscedasticity (see Fig. 1). As a consequence, the power of all four test statistics at $\rho_{V}=0$ is lower than the power under homoscedasticity when the number of studies is relatively large, due to this altered publication bias mechanism.

## 5 Discussion and conclusion

The performances of the Egger test, the rank correlation test, the weighted DL test and the weighted REML test were investigated for testing publication bias in the presence of residual heteroscedasticity for mean differences. Residual heteroscedasticity for mean differences is plausible and realistic, since it represents heterogeneity in inter-participant variability across studies. Indeed, participants can be more alike in one study than in another study, depending on the implemented selection criteria for the study participants. For instance, pragmatic clinical trials may hardly use any selection criteria, since they focus on efficiency, while other clinical trials may focus on efficacy on a specific set of participants with a particular symptom or disease in a limited age range. Note that the form of heteroscedasticity that we included has been studied in more sophisticated ways in the area of multilevel models [20].

In the simulation study, we found the Type I error of the Egger's test inflated. This is a well-known phenomenon for log odds ratios [31] and standardized mean differences [32,33], but in our simulation such inflation is unexpected. The cause is related to the way we simulated the data. We have chosen to simulate data based on an IPD model, which is different from simulations based on aggregated data model that other researchers have used to study performances of tests for publication bias [22,24,28]. In our simulation, the distribution of sample size $n_{i}$ affects Egger's test directly. If we select the overdispersion parameter $\gamma_{i}\sim \Gamma \left(1/3,3\right)$ in $n_{i}|\gamma_{i}\sim \text{Poi}\left(\lambda exp\left\{0.5\gamma_{i}\right\}\right)$, the distribution of $n_{i}$ is less extreme or less skewed than with $\gamma_{i}\sim \Gamma \left(1,1\right)$ and the Type I error rate for Egger's test reduces to 12.1 (for m=50). One very large study in a meta-analysis becomes an influential point in Egger's regression analysis that strongly affects the test on intercept, especially when heterogeneity in study effect sizes are not considered as in the weighted regression approaches.

Heteroscedasticity renders the four tests unreliable in testing for publication bias in meta-analysis. It causes the Type I error to deviate from the nominal level substantially and also from the level obtained under homoscedasticity if the test was not nominal (Egger's test). For the Egger test, the introduction of heteroscedasticity increases the variability in $1/S_{i}$. Together with the correlation between the heteroscedasticity $V_{i}$ and heterogeneity $U_{i0}-U_{i1}$, the standard error of the intercept in the regression model reduces, leading to more rejections of the null hypothesis than under homoscedasticity. For the weighted regression approaches something similar is going on. The correlation between the heteroscedasticity and heterogeneity induces a correlation between $D_{i}$ and $S_{i}$, which causes an increased Type I error rate. For the rank-correlation test, introducing heteroscedasticity causes the Type I error rate to drop below the nominal level, but the larger the number of studies the smaller the effect of heterogeneity. The introduction of heteroscedasticity reduces the variance of Kendall's tau statistic, thereby reducing the variance of the test statistic. This results in a non-standard normal distribution with a variance less than one, introducing the conservative Type I error rates.Furthermore, it decreases the power strongly in most cases. Tests for publication bias have always shown low statistical power even in the absence of heteroscedasticity [3,28,29,33]. With this known evidence and our newly presented criticism, testing for publication bias in meta-analysis with continuous outcomes should be avoided.

We did not include any of the publication bias methods (e.g., trim and fill, Copas’ selection method) that could correct the pooled estimate from a meta-analysis [9,22]. These estimation approaches would typically make use of the correlation between the study effect size and its standard error in one way or another, as do the test statistics we investigated. Since heteroscedasticity is destroying this relationship, we expect that these correction methods would not be able to correct the pooled estimate appropriately. As we have demonstrated, diagnosing publication bias using the correlation between effect size and standard error, is strongly affected.

Heteroscedasticity also affected the mechanism of publication bias, making it difficult to disentangle these two mechanisms at an aggregated level. Thus when heteroscedasticity is anticipated from the topic of study, it may be recommended to collect and pool the individual participant data. The heteroscedasticity can then be potentially modeled, although it remains unknown how to address the publication bias into this approach. More research is needed to be able to model the correlation between study effect sizes and its standard error.

## Footnotes

1 In case the weight is changed to $1/S_{i}^{2}$, the weighted regression approach is identical to Egger's test.

2 In the random effects model it is often assumed that the random variables $U_{i}$ and $\varepsilon_{i}$ are independent and normally distributed, but due to our random heteroscedastic variable $exp\left(V_{i}\right)$ both assumptions will be violated.
